# The Life Experience of Patients after the Implantation of Cardiovascular Implantable Electronic Devices: A Qualitative Meta-Synthesis

**DOI:** 10.31083/j.rcm2410304

**Published:** 2023-10-23

**Authors:** Xin-yi Zhou, Qi-qi Ke, Ju-kun Su, Ke Hu, Qiao-hong Yang

**Affiliations:** ^1^School of Nursing, Jinan University, 510632 Guangzhou, Guangdong, China

**Keywords:** cardiovascular implantable electronic devices, life experience, qualitative research, meta-synthesis

## Abstract

**Background:**

Cardiovascular implantable electronic devices (CIED) are more and more widely used in the clinical treatment of cardiovascular diseases. However, CIED implantation may also result in a variety of physical, psychological, and social problems among patients. To help patients adapt to life after CIED implantation, it is important to know patients’ needs from their perspectives. Explore the needs of CIED patients from their perspectives to guide healthcare providers to improve their quality of life.

**Methods:**

PubMed, Web of Science, Embase, the Cochrane Library, CNKI, the VIP database, the Wanfang database, and the China Biomedical Literature database were searched for qualitative studies on the experience of patients with CIED dating from January 2000 to August 2022. The quality of each article was evaluated according to the 2016 edition of the Joanna Briggs Institute Evidence-Based Health Care Center Qualitative Research Quality Evaluation Criteria and an integrative meta-synthesis was undertaken.

**Results:**

A total of 18 documents were included, and 111 categories were extracted. Analysis of the data resulted in the identification of 3 themes and 12 subthemes. The first theme, Equipment Symbiosis, included “Mixed feelings about the device as part of the body”, “Mixed feelings about the patient’s role”, and “Mixed feelings about an electrical stimulus”. The second theme, External Support, included “Husband and wife relationship damaged”, “Eager to participate, unwilling to be overprotected”, “Want to return to work but are forced to leave”, and “Information supply and demand mismatch”. The third theme, Self-coping, included “How to face a doctor”, “How to deal with activity restrictions”, “How to face yourself”, “How to face the future”, and “How to face death”.

**Conclusions:**

Healthcare providers need to accelerate technological innovation and clinical adoption of CIED. Additionally, healthcare providers need to establish a diverse support system led by medical staff, with family members, peers, and society working together, and improve CIEDs remote monitoring to help patients improve their quality of life.

## 1. Introduction

Cardiovascular disease (CVD), primarily including arrhythmia, heart failure, and coronary heart disease, is the leading cause of death globally [1]. The World Health Organization estimates that nearly 17.9 million people die from CVD each year, accounting for approximately 32% of the total number of deaths worldwide [1]. Treatment options include traditional drug therapy, surgical treatment, and, more recently, device therapy.

Interventional therapy is the fastest-growing and most promising treatment modality for the prevention and treatment of CVD and is currently recommended as a primary prevention method for related conditions [2, 3]. Cardiovascular implantable electronic devices (CIEDs) include pacemakers (PMs), implantable cardioverter-defibrillators (ICDs), and cardiac resynchronization therapy (CRT) devices [4]. The implantation of CIEDs is the only effective means of rapidly diagnosing and treating life-threatening ventricular arrhythmias, and it is also one of the most effective methods for treating heart failure and preventing sudden cardiac arrest [5]. In a survey covering 61 countries, of 1,002,664 CIEDs, 75% were new implants and the remainder were replacements [6]. Another survey showed that from 2002 to 2020, the CIED implantation rate in the UK increased fourfold [7, 8]. The rate of CIED implantation continues to increase, as does its application in the clinical treatment of CVD. Studies have found that implanting a CIED early in the course of CVD-related disease can reduce morbidity and mortality by 30% to 54% and significantly prolong the survival time of patients [9, 10]. However, in contrast to its therapeutic role, CIED implantation may also result in a variety of physical, psychological, and social problems among patients.

Patients with a CIED can experience physical discomforts such as pain, infection, sleep disturbance, and memory loss [11, 12, 13], as well as negative emotions such as fear of electric shock, self-doubt, fear of death, and worry about the future [10, 14, 15, 16]. CIEDs also restrict patients’ activities, including sex, driving, and socializing [12, 17, 18, 19], and patients may face problems such as body dysmorphia and involuntary job transfers, all of which seriously reduce their quality of life [17, 18]. Notably, the occurrence of emotional disorders is an important risk factor for CVD and the recurrence of cardiac events [20]. Therefore, it is particularly important to pay attention to the life experience of patients with CIEDs and understand their support needs to allow the formulation of specific and effective interventions to help patients adapt to life after CIED implantation.

Quantitative research can objectively reflect the quality of life of patients with CIEDs through scale evaluation, but cannot be used to appraise the life experience of patients. Although studies have employed qualitative methods to understand the life experience of patients after the implantation of CIEDs, owing to the influence of demographic factors, medical level, cultural background, and regional differences, the results of a single qualitative study cannot fully and reliably reflect the life experience of patients after CIED implantation. Instead, qualitative synthesis, which guides clinical practice and future research by integrating the qualitative evidence obtained from the existing literature, must be undertaken. A qualitative synthesis also cannot fully and reliably reflect the life experiences of patients after CIED implantation, however, it helps. The combined results can help healthcare providers formulate interventions tailored to patients’ needs and expectations, while also allowing the detection of important aspects of the experiences of patients that are currently unaddressed. Therefore, this study aimed to gain an in-depth understanding of the life experience of patients after CIED implantation through meta-synthesis and provide guidance for improving the quality of life of these patients.

## 2. Materials and Methods

### 2.1 Search Strategy and Selection

English databases (PubMed, Web of Science, Embase, and the Cochrane Library) and Chinese databases (CNKI, the VIP database, the Wanfang database, and the Chinese Biomedical Literature database) were searched for qualitative research on the life experience of patients after implantation of cardiovascular electronic devices. Although the first CIED was implanted in 1958 [21], with the development of medical technology and services, patients are currently facing completely different problems compared with patients implanted decades ago. Therefore, this study examines relevant papers published since the 21st century, with a specific period from January 2000 to August 2022. A combination of Medical Subject Headings (MeSH) terms and keywords, such as “Pacemaker, Artificial”, “Defibrillators, Implantable”, “Cardiac Resynchronization Therapy Devices”, “Life”, “Feel”, “Experience”, and “Qualitative Research” were used to conduct a comprehensive search of the above-mentioned databases. The retrieval strategies are shown in Table 1, using PubMed as an example. The current review was undertaken following the Preferred Reporting Items for Systematic Reviews and Meta-Analyses (PRISMA) guidelines.

**Table 1.** **PubMed search strategy**.

Database	Search	Query	Results
PubMed	#1	(Pacemaker, Artificial[MeSH Terms]) OR (Defibrillators, Implantable[MeSH Terms]) OR (Cardiac Resynchronization Therapy Devices[MeSH Terms]) OR (Artificial Pacemaker[Title/Abstract]) OR (Artificial Pacemakers[Title/Abstract]) OR (Pacemakers, Artificial[Title/Abstract]) OR (Cardiac Pacemaker, Artificial[Title/Abstract]) OR (Artificial Cardiac Pacemaker[Title/Abstract]) OR (Artificial Cardiac Pacemakers[Title/Abstract]) OR (Cardiac Pacemakers, Artificial[Title/Abstract]) OR (Pacemaker, Artificial Cardiac[Title/Abstract]) OR (Pacemakers, Artificial Cardiac[Title/Abstract]) OR (Defibrillator, Implantable[Title/Abstract]) OR (Implantable Defibrillator[Title/Abstract]) OR (Implantable Defibrillators[Title/Abstract]) OR (Implantable Cardioverter-Defibrillators[Title/Abstract]) OR (Implantable Cardioverter Defibrillator[Title/Abstract]) OR (Cardioverter Defibrillator, Implantable[Title/Abstract]) OR (Cardioverter Defibrillators, Implantable[Title/Abstract]) OR (Defibrillator, Implantable Cardioverter[Title/Abstract]) OR (Defibrillators, Implantable Cardioverter[Title/Abstract]) OR (Implantable Cardioverter Defibrillators[Title/Abstract]) OR (Cardioverter-Defibrillators, Implantable[Title/Abstract]) OR (Cardioverter-Defibrillator, Implantable[Title/Abstract]) OR (Implantable Cardioverter-Defibrillator[Title/Abstract]) OR (Cardiac Resynchronization Therapy Device[Title/Abstract]) OR (Biventricular Pacemakers, Artificial[Title/Abstract]) OR (Artificial Biventricular Pacemaker[Title/Abstract]) OR (Artificial Biventricular Pacemakers[Title/Abstract]) OR (Biventricular Pacemaker, Artificial[Title/Abstract]) OR (Pacemaker, Artificial Biventricular[Title/Abstract]) OR (Pacemakers, Artificial Biventricular[Title/Abstract]) OR (Cardiovascular Implantable Electronic Devices[Title/Abstract])	49,195
	#2	(Life*[Title/Abstract]) OR Live*[Title/Abstract]) OR Experience*[Title/Abstract]) OR (Feel*[Title/Abstract]) OR (Need*[Title/Abstract]) OR (Attitude*[Title/Abstract]) OR (View*[Title/Abstract])	3,760,574
	#3	Qualitative Research[MeSH Terms] OR (Research, Qualitative[Title/Abstract]) OR (Qualitative Study[Title/Abstract]) OR (Interviews[Title/Abstract]) OR (Phenomenological Research[Title/Abstract]) OR (Phenomenological Study[Title/Abstract]) OR (Phenomenon[Title/Abstract]) OR (Grounded Theory[Title/Abstract]) OR (Theory, Grounded[Title/Abstract]) OR (Ethnographic Research[Title/Abstract]) OR (Ethnographic Study[Title/Abstract]) OR (Ethnography [Title/Abstract]) OR (Case Research[Title/Abstract]) OR (Case Study[Title/Abstract]) OR (Action Research[Title/Abstract]) OR (Action Study[Title/Abstract]) OR (Narrative[Title/Abstract])	629,506
	#4	#1 AND #2 AND #3	194

### 2.2 Inclusion and Exclusion Criteria

According to the PICoS principles, the inclusion criteria of papers were set as follows: (1) P (participant): aged 18 or over patients with a CIED; (2) I (phenomenon of interest): life experience of patients after CIED (PM, ICD, CRT) implantation; (3) Co (context): patient’s home or workplace, hospital or clinic; (4) S (study design): qualitative research, including phenomenological research, grounded theory, case study, ethnography, and action research. The exclusion criteria were as follows: (1) mixed population studies, qualitative data cannot be separated; (2) full text unavailable, incomplete data, and duplicate publications; (3) studies that were not in Chinese or English.

### 2.3 Data Extraction 

Two authors independently screened the articles, extracted the data, and cross-checked them in strict accordance with the inclusion and exclusion criteria. In case of disagreement, a third party assisted in the decision. When screening articles, duplicate documents were first removed using EndNote document management software. Subsequently, the title and abstract of each article were read and, after excluding irrelevant documents, the full text was read to determine whether an article was finally included in the analysis. The data extracted mainly included the author (country) year, the qualitative research method used, the research object, the phenomenon of interest, the location, and the main results.

### 2.4 Quality Assessment

Two authors independently evaluated the included articles according to the 2016 edition of the JBI Evidence-based Health Care Quality Evaluation Standards for Qualitative Research [22]. Using these criteria, each item is rated as “yes”, “no”, or “unclear”. If the criteria are fully met, then the possibility of bias is minimal, and the item is classified as grade A. If some of the above quality standards are met, then the possibility of bias is moderate, and the item is classified as grade B. The items that do not meet any of the above quality standards have a high possibility of bias and are classified as grade C. Once the quality of the articles had been independently evaluated, the results of the two authors were compared. In case of disagreement, the two authors sought to reach a consensus or asked a third party to decide whether to include the article after arbitration. Finally, studies with quality grades A and B were included, while those graded C were excluded.

### 2.5 Data Synthesis

Thomas and Harden’s thematic and content analysis methodology of synthesising qualitative studies was used to guide this meta-synthesis [22]. Before the synthesis, the included studies were read and reread by two authors to obtain a preliminary understanding. According to the first stage of thematic and content analysis, all results and findings were inductively coded line-by-line according to their meaning and content. In the second stage, these codes were grouped by comparing their similarities and differences to create descriptive themes. Finally, reread descriptive themes, and new conceptions, understandings, or assumptions were identified. In this stage, analytical findings (themes and subthemes) were generated that presented the key findings of the primary studies. The initial coding process was carried out by two authors. The identification of emergent descriptive themes and analytic themes was completed by one author. The data analysis process was subsequently checked by the whole research team to ensure the congruence of the interpretations and the adequacy of the analytic themes.

## 3. Results

### 3.1 Summary of Findings

**Fig. 1.**
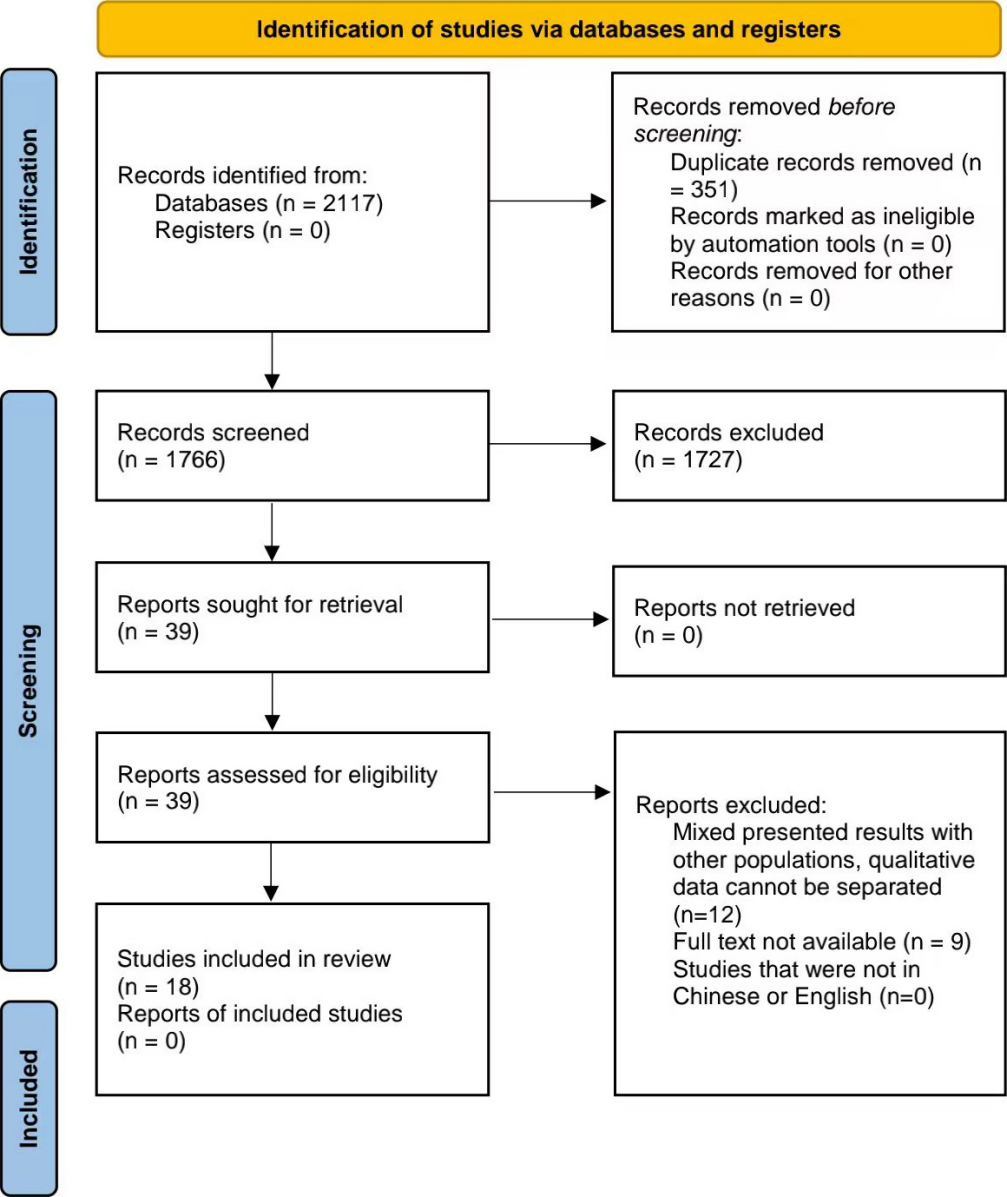
**Flowchart of the included studies**.

As shown in Fig. 1 and Table 2 (Ref. [10, 11, 12, 13, 14, 15, 16, 17, 18, 19, 23, 24, 25, 26, 27, 28, 29, 30]), a total of 2117 articles were retrieved from the databases, and 18 were finally included after deduplication and screening [10, 11, 12, 13, 14, 15, 16, 17, 18, 19, 23, 24, 25, 26, 27, 28, 29, 30]. Publication dates ranged from 2000 to 2022. The largest number of studies (*n* =  4, 22.22%) was from Sweden, the UK, the USA, and Italy each had two studies, Australia, Netherlands, Norway, Spanish, China, Iran, Singapore, and Turkey each had one study. Across the articles, there were 301 patients. Their age ranged from 21 to 93 years old. Among them, 207 (68.77%) were male, 94 (31.23%) were female, 194 (64.45%) were married, 47 (15.61%) were single and others, the marital status of 60 (19.94%) people is unclear, 248 (82.39%) had ICD, 36 (11.96%) had PM, 17(5.65%) had cardiac resynchronization therapy-defibrillator (CRT-D). Of the 18 articles included, 7 were phenomenological studies [10, 12, 13, 14, 18, 23, 27], 5 were descriptive qualitative studies [11, 16, 24, 26, 28], 4 were grounded theory studies [15, 17, 19, 29], 1 was interpretive interactionism study [25], and 1 was deductive exploratory study [30].

**Table 2.** **Basic characteristics of the articles (*n* = 18)**.

Article	Country	Year	Qualitative research method	Research object (total number of cases, male/female)	Phenomenon of interest	Location	Main results
Fridlund *et al*. [13]	Sweden	2000	Phenomenological research; semi-structured interviews	Patients with an ICD (15, 10/5)	Perceptions of living conditions among patients living with an ICD	Patient’s home	Six themes: Security, gratitude, presence, networking, faith in the future, gaining awareness.
Tagney *et al*. [16]	UK	2003	Descriptive qualitative research; semi-structured interviews	Patients with an ICD (8, 6/2)	Patient’s experience of living with an ICD	Patient’s home	Three themes: Non-individualised nature of information, adjustments to living with the device, future outlook.
							Three unique findings identified: Concealment of problems and symptoms, funding issues, and inability to access appropriate support and advice.
Kamphuis *et al*. [24]	Netherlands	2004	Descriptive qualitative research, semi-structured interviews, content analysis	Patients with an ICD (21, 12/9)	How ICD recipients perceive their lives during the first year after implantation of the device	Patient’s home	Seven major categories: physical deterioration, cognitive changes, perceived social support, dependency, contact with the doctor, confrontation with mortality and uncertainty surrounding having a shock.
Anderson *et al*. [25]	Australia	2004	Interpretive interactionism research, thematic and content analysis	War veteran with a pacemaker (8, 8/0)	How does the war veteran experience his body in relation to invasive cardiac technology	Patient’s home	Five themes: emotional knowing; the medical encounter; belief in the myth of miracle; technological constraint; and the altered heart.
Bolse *et al*. [23]	USA	2005	Phenomenological research; semi-structured interviews	Patients with an ICD (14, 8/6)	How patients with an ICD perceive their living situation	Patient’s home	Three themes: Trust, adaptability, empowerment.
Malm *et al*. [15]	Sweden	2006	Grounded theory; semi-structured interviews	Patients with a pacemaker (13, 6/7)	Daily life experience of patients with a pacemaker	Patient’s home or workplace	Two themes: Perceived social engagement, emotional state.
McDonough *et al*. [26]	USA	2009	Descriptive qualitative research	Patients with an ICD (20, 8/12)	Experiences and concerns of young adults living with an ICD	Telephone and Internet interviews	Four categories: psychosocial, developmental, physical, and economical.
							Six themes: returning to normal, mood disturbances, body image concerns, childbearing and childrearing, expectation regarding physical activity, financial security.
Morken *et al*. [17]	Norway	2010	Grounded theory; semi-structured interviews	Patients with an ICD (16, 11/5)	Life experience of ICD recipients	Hospital or patient’s home	One core category: Reconstructing the unpredictability of living with an ICD.
							Four specific categories: Loss of control, regaining control, lack of support, seeking support.
Flemme *et al*. [19]	Sweden	2011	Grounded theory	Patients with an ICD (16, 9/7)	Key concerns of people with ICDs and how they deal with them in everyday life	Patient’s home, university, or office	One core category: Incorporating uncertainty into everyday life.
							Four specific categories: Restriction of activities, distraction, acceptance of being an ICD patient, reassessment of life.
Palacios-Ceña *et al*. [27]	Spanish	2011	Phenomenological research	Male ICD recipients 18 years of age or older (22, 22/0)	Determine the experience of Spanish implantable defibrillator recipients	Patient’s home	Seven themes: accepting the change, developing strategies, rethinking your relationship with your partner and rather more emotionally distant, giving up some of your independence, transformed, with life insurance, in a state of continual uncertainty and waiting.
Humphreys *et al*. [28]	UK	2016	Descriptive qualitative research , semi-structured interviews; thematic analysis	Patients with an ICD (18, 11/7)	Lived experiences of ICD recipients	Patient’s home	Three themes: physical consequences, emotional consequences, coping with the ICD.
							Five subthemes: feeling vulnerable and uncertain, anxiety and depression, avoidance/restrictive behaviors, acceptance, concealment.
Xuan *et al*. [12]	China	2017	Phenomenological research; semi-structured interviews; content analysis	Patients with a permanent pacemaker (15, 9/6)	Real-life experiences and feelings of a patient with a permanent pacemaker	Unclear	Three themes: Pacemaker distress, pacemaker adaptation, pacemaker care needs.
							Eight subthemes: Anxiety and worry, physical dysfunction, social impairment, sexual impairment, mandatory pacemaker acceptance, pacemaker satisfaction, home care needs, continued care needs.
Dehghanzadeh *et al*. [29]	Iran	2018	Grounded theory	Patients with heart failure who had a CRT-D (17, 9/8)	Patients’ experiences of living with cardiac resynchronization therapy (CRT-D)	Hospital	Five themes: Losing integrity, Attempting to cope with cardiac resynchronization therapy defibrillator, Coexisting, Outcomes: From frustration to empowerment, Barriers and facilitators to living with cardiac resynchronization therapy defibrillator.
Garrino *et al*. [14]	Italy	2018	Phenomenological research; semi-structured interviews	Patients with an ICD (20, 15/5)	Daily experiences of patients living with an ICD	Hospital	Four themes: Living in fear, relying on technology, understanding the ICD and how to live with it, coping with the impact of an ICD on everyday life.
Ooi *et al*. [11]	Singapore	2018	Descriptive qualitative research; thematic analysis; semi-structured interviews	Patients with an ICD (16, 13/3)	The perceptions of quality of life, coping strategies, and learning needs among patients living with an ICD	Hospital	Seven themes: Experiencing ICD shock, ambiguous “love-hate” relationship with the ICD, receiving support from healthcare professionals and social networks, gaining acceptance and returning to normal, physical coping, emotional coping, need for education.
Rosi *et al*. [18]	Italy	2021	Phenomenological research; semi-structured interviews	Patients with an ICD (16, 13/3)	Lived experiences of people with ICDs	Hospital	Four themes: “My Heart is Asleep”; “But what’s the Thing…”; “I Can’t Hug the Microwave”; “With this, I’m Well for the Rest of my Life”.
Sert *et al*. [10]	Turkey	2021	Phenomenological research; semi-structured interviews	Patients with an ICD (26, 19/7)	The impact of ICDs on patients’ lives and their experience of worrying about death	Hospital	Three themes: Physical impact of the device on the body, social impact in daily life and adaptation to restrictions, seeking social support.
							Ten subthemes: Changes in body function, like a part of the body, necessary to live, limiting behaviors of everyday life, seeking social support, inescapable truths, moving on a sure path, trust in life insurance.
Wising *et al*. [30]	Sweden	2022	Deductive exploratory research, semi-structured interviews; deductive thematic analysis	Patients over 80 years of age with ICD (20, 18/2)	Octogenarians’ experience, knowledge, and attitude of living with an ICD	Patient’s home	Three themes: Life goes on, Their Health; The Janus-Faced Device, Their attitudes; and Mind the gap, Their knowledge.

ICD, implantable cardioverter-defibrillator; CRT-D, cardiac resynchronization therapy-defibrillator.

### 3.2 Quality Assessment

As shown in Table 3 (Ref. [10, 11, 12, 13, 14, 15, 16, 17, 18, 19, 23, 24, 25, 26, 27, 28, 29, 30]), the methodological quality evaluation results showed that 3 articles were grade A and 15 were grade B. Notably, only three studies clarified the potential influence of the researchers’ beliefs and values on the research, and only eight studies addressed the researchers’ influence on the research and the influence of the research on the researchers. In addition, ethical considerations were unavailable for one study.

**Table 3.** **Methodological quality evaluation results of the included articles (*n *= 18)**.

Article	(1)	(2)	(3)	(4)	(5)	(6)	(7)	(8)	(9)	(10)	Grade
Fridlund *et al*. [13]	Yes	Yes	Yes	Yes	Yes	No	No	Yes	Yes	Yes	B
Tagney *et al*. [16]	Yes	Yes	Yes	Yes	Yes	No	Yes	Yes	Yes	Yes	B
Kamphuis *et al*. [24]	Yes	Yes	Yes	Yes	Yes	No	No	Yes	Yes	Yes	B
Anderson *et al*. [25]	Yes	Yes	Yes	Yes	Yes	No	No	Yes	Yes	Yes	B
Bolse *et al*. [23]	Yes	Yes	Yes	Yes	Yes	No	Yes	Yes	Yes	Yes	B
Malm *et al*. [15]	Yes	Yes	Yes	Yes	Yes	No	No	Yes	Yes	Yes	B
McDonough *et al*. [26]	Yes	Yes	Yes	Yes	Yes	No	No	Yes	Yes	Yes	B
Morken *et al*. [17]	Yes	Yes	Yes	Yes	Yes	No	Yes	Yes	Yes	Yes	B
Flemme *et al*. [19]	Yes	Yes	Yes	Yes	Yes	No	No	Yes	Yes	Yes	B
Palacios-Ceña *et al*. [27]	Yes	Yes	Yes	Yes	Yes	Yes	Yes	Yes	Yes	Yes	A
Humphreys *et al*. [28]	Yes	Yes	Yes	Yes	Yes	No	Yes	Yes	Yes	Yes	B
Xuan *et al*. [12]	Yes	Yes	Yes	Yes	Yes	No	Yes	Yes	Unclear	Yes	B
Dehghanzadeh *et al*. [29]	Yes	Yes	Yes	Yes	Yes	No	No	Yes	Yes	Yes	B
Garrino *et al*. [14]	Yes	Yes	Yes	Yes	Yes	No	No	Yes	Yes	Yes	B
Ooi *et al*. [11]	Yes	Yes	Yes	Yes	Yes	Yes	Yes	Yes	Yes	Yes	A
Rosi *et al*. [18]	Yes	Yes	Yes	Yes	Yes	No	No	Yes	Yes	Yes	B
Sert *et al*. [10]	Yes	Yes	Yes	Yes	Yes	No	No	Yes	Yes	Yes	B
Wising *et al*. [30]	Yes	Yes	Yes	Yes	Yes	Yes	Yes	Yes	Yes	Yes	A

Notes: (1) Were the stated philosophical views consistent with the research methods. (2) Were the research methods consistent with the research questions or objectives. (3) Were the research methods consistent with the data collection methods. (4) Were the research methods consistent with the data and were the analysis and expression methods consistent. (5) Were the research methods and the result interpretations consistent. (6) Was the potential influence of the researcher’s concept and values on the research clarified. (7) Were the researcher’s influence on the research and the impact of the research on the researchers explained. (8) Did the results fully represent the stated meaning of the participants. (9) Did the research conform to the current ethical standards and was it accompanied by a research ethics approval certificate recognized by academic institutions. (10) Did the conclusions of the research match the data analysis and interpretation.

### 3.3 Themes

Twelve key subthemes were extracted from the eighteen included studies. Through meta-synthesis, three themes were identified by comparing and contrasting the twelve subthemes. The lived experiences of patients implanted with CIEDs are categorized according to the following three themes: (1) equipment symbiosis; (2) external support; (3) self-coping. Both the descriptive and analytical findings are reported in the following sections.

#### 3.3.1 Theme 1. Equipment Symbiosis

The theme describes the complex feelings of patients with CIED symbiosis. This theme includes three subthemes: mixed feelings about the device as part of the body; mixed feelings about the patient’s role; and mixed feelings about electric shock.

Subtheme 1. Mixed Feelings about the Device as Part of the Body

For the patient, the CIED is both a foreign body and a part of the body. Some patients see the CIED as a foreign body that has invaded them, causing physical disturbances such as pain, changes in appearance, difficulty falling asleep, and memory loss. However, other patients consider the CIED to be a part of their body and are grateful for it.

“It’s invasive” [25].

“Severe pain like an ant bite […] yes, it is part of my body” [11].

“As soon as you take off your shirt, everyone will see the bomb below” [18].

“My short-term memory is gone […] I am grateful for the opportunity to have an ICD implanted and I thank the hospital for providing me with this assistance” [13].

“I don’t try and cover it up at all, it’s part of who I am” [28].

Subtheme 2. Mixed Feelings about the Patient’s Role

For patients, having a CIED implanted not only reminds them of their illness but also helps them fight the disease. Some patients believe that having a CIED implanted is a reminder that they are sick, and feel depressed. However, other patients feel that the implanted CIED is helping them fight their disease and feel safe and reassured.

“I feel violated because I can’t do things the way I used to. I have to think ahead, do I have the strength to go there now? The ICD is reminding me that I am sick” [13].

“I feel safer than in the hospital because when I need an electrical stimulus in the hospital, it takes time for the nurse to arrive; however, with the ICD, the stimulus is immediate” [10].

Subtheme 3. Mixed Feelings about the Electrical Shock

The patient is terrified of an electric shock but still intentionally triggers one. The CIED will automatically discharge to save the patient’s life but at the expense of a strong shock to the patient, or multiple shocks. Shocks are unpredictable and can cause fear even in CIED patients who have never experienced an electric shock. Nevertheless, the patients were curious about their endurance, and some chose to deliberately trigger the device to cause an electric shock.

“I felt like I was hit by a freight train […] I trigger this thing on purpose because I want to know what it will feel like, to see what I can do” [16].

“I’ve never had any reaction to a single shock, but if you get two or three in a row, you’re probably dead […] I don’t have any warning, it happens so fast” [17].

“If it did happen, I hope I’m in a coma” [14].

#### 3.3.2 Theme 2. External Support

This theme describes the current state of the patient’s external support and the patient’s support needs. This theme includes four subthemes: husband and wife relationship damaged; eager to participate, unwilling to be overprotected; want to return to work but are forced to leave; information supply and demand mismatch.

Subtheme 1. Husband and Wife Relationship Damaged

Even if the partner is strong enough to face the patient’s condition, the marital relationship can still be negatively affected. The partner provides strong emotional support to the patient and faces the disease together with the patient. However, the patients’ intense emotional needs and sexual barriers can make them feel stressed. In addition, patients sometimes hide the fact that they have a CIED for fear of their partners worrying, leading to a gradual breakdown in communication between husband and wife.

“Throughout this time period, my wife consistently supported me. Her presence and help hearten me” [29].

“I’m very good at talking about anxiety, but then my husband went to see a therapist because he said he was empty and couldn’t answer all my questions” [17].

“We haven’t had sex for a long time, and I feel that our relationship has begun to fade” [12].

“I didn’t say anything to my wife, I didn’t want her to panic” [16].

Subtheme 2. Eager to Participate, Unwilling to Be Overprotected

The support of relatives and friends makes patients feel content; however, overprotection by relatives and friends will not only increase their burden but also makes patients dependent while also longing for independence. The only recourse for patients is to act as if they have recovered, to do what they used to do before CIED implantation, but this is often not always possible. Additionally, patients who are truly alone and are forced to handle everything independently will feel frustrated because they do not have the support of relatives and friends.

“I am happy to live with my sister […] I don’t want to swim without someone to accompany me” [23].

“My family said, oh, you can’t do that, but I know I can” [16].

“Because they worried that I was doing too much strenuous work, I had to cut down on gardening a little bit” [15].

“I am not married, I must learn to live independently” [10].

Subtheme 3. Want to Return to Work but Are Forced to Leave

Patients hope for a smooth return to work but most of them face early retirement or dismissal. When a patient receives a CIED, the employer feels that they are no longer qualified for their jobs, and the patient faces early retirement or dismissal. Patients also do not have the confidence to change jobs. Not being able to return to work smoothly not only reduces the patient’s quality of life but also leaves them devastated.

“They don’t think I can come back because the work equipment interferes with my equipment […] I want to apply for other jobs, but I’m not sure if the employer will accept me if I tell the truth” [16].

“The loss of my job affected me. It made me feel worthless” [27].

“Now I can’t be too tired physically, and I need another assistant to help me, which will mean my wages will be reduced” [11].

Subtheme 4. Information Supply and Demand Mismatch

Patients want to be informed of the prognosis following the implantation of the CIED but often doctors instead provide a lot of technical information about the CIED. Patients initially lacked awareness of what was happening to them and why the ICD was implanted, but they will use metaphors to describe how their heart works. Despite the technical information provided by physicians, patients were not satisfied because their information needs for post-implantation preparation for daily living, sports rehabilitation, and device discontinuation were not met [11, 16, 17].

“They implanted this in me because they said my heart was asleep […] I have this friend who can help me when I need it” [18].

“They explained in detail what it was, where it was, how big it was, and how long the battery was expected to last” [14].

“I was never asked how I was feeling or offered any kind of supportive dialogue. It made me very unsatisfied” [13].

#### 3.3.3 Theme 3. Self-Coping

This theme describes two attitudes of how the patient himself confronts the doctor, activity limitations, self, future, and death. This theme includes five subthemes: how to face a doctor; how to deal with activity restrictions; how to face yourself, how to face the future; how to face death.

Subtheme 1. How to Face a Doctor

Most patients have absolute trust in doctors but sometimes choose to ignore from them. The inability of patients to cope with the disease on their own makes them trust the doctor. However, when the doctor’s advice is inconsistent with the patient’s cognition, the patient will choose to ignore the doctor’s advice and conceal his condition from the doctor.

“Doctors know how to deal with my disease, but I don’t know what to do, I trust the doctor” [18].

“They said I could live a normal life. But I was miserable and insecure” [17].

“The traffic law requires a patient like me to avoid driving for a year, but the doctor said that if I want to, I could drive properly, but I don’t think I can […] I thought I had a small injury, so I didn’t tell them” [16].

Subtheme 2. How to Deal with Activity Restrictions

Following CIED implantation, patients impose constraints on themselves to avoid electromagnetic shocks, and even give up activities they used to enjoy but now require too much energy. This leads to negative emotions and a perceived loss of quality of life. Therefore, some patients choose to reduce activity restrictions to alleviate negative emotions, but others over-restrict activities due to fear of disease progression, and even change dietary habits.

“I lost the will to do anything” [16].

“My quality of life has declined because they took away my driver’s license” [17].

“They also told me to quit smoking and change my diet, but I didn’t make any changes […] I don’t eat cabbage anymore, nor grapefruit and pineapple” [14].

Subtheme 3. How to Face Yourself

Patients oscillate between self-doubt and self-acceptance. Self-doubt arises when patients experience negativity and social prejudice. However, they will reassess their lives and accept themselves through strategies such as finding themselves, changing life goals, changing methods, diverting attention, planning carefully, and participating in support groups [13, 15, 19, 23].

“I dare not be like before, I am cowardly” [13].

“The people at the blood donation center asked me, do you think we will accept blood from second-class people […] I can no longer fish like before, but I can repair fishing equipment for others” [15].

“I think it helps to be around patients of similar age and occupation” [23].

Subtheme 4. How to Face the Future

Patients are both worried and confident about the future. Their worries mainly stem from uncertainties and unknowns after implantation, including equipment, batteries, genetics, life expectancy, children, and funds [10, 14, 16, 18]. Therefore, they want to have access to a continuum of medical care so that the problems they experience after discharge can be addressed in a timely manner. The confidence of patients in the future stems from the joy of being reborn, their trust in the equipment, and their confidence in technological progress.

“I would feel restless because of a dead battery […] I didn’t know what was going to happen, I hope to get help when I get out of the hospital too” [10].

“I have two children, there is a chance one of my children will have this and that makes me furious” [26].

“I don’t know how long I’ll live […] I don’t know if I have enough money” [16].

“Now I can move around freely again, and all my previous lethargy has disappeared” [15].

“If my heart stops beating, the device starts and it goes all out” [19].

“It will keep improving, I wish there was a smaller defibrillator” [18].

Subtheme 5. How to Face Death

Patients fear death but most can face it calmly. All the patients believe that they are living on the borderline between life and death. The implantation of a CIED makes patients feel closer to death or farther from it. Some patients avoid talking about death because they are afraid of it, whereas others can face death calmly and live actively.

“… the risk of becoming a vegetable might be greater when you have an ICD than when you do not because then you might die immediately” [30].

“I can’t stop doing things because I’m afraid of death, I have to make the most of each day and stay positive” [17].

## 4. Discussion

This study synthesized the results of eighteen qualitative studies on the lived experience of CIED patients, provided information focusing on three aspects of device symbiosis, external support, and self-coping, and revealed important aspects of the patient experience that are currently unaddressed, namely, that is, patients’ needs for improved equipment, precise external support and continuous medical care services. It can help healthcare providers formulate interventions tailored to patients’ needs. It is worth noting that the themes often include polar opposite feelings and opinions, and cover some rich, complex, aspects of lived experience. This suggests the following four important aspects. First, different patients have different life experiences. This suggests that healthcare providers should provide personalized medical care based on patient needs. Second, some patients have a good life experience and some have a bad life experience. This suggests that the bad life experience of patients is not absolute. Healthcare providers may be able to help patients improve their quality of life through interventions and support to change their life experiences for the better. Third, CIED implantation has complex effects on multiple aspects of a patient’s life. This shows that healthcare providers should pay attention to the life experience of patients after CIED implantation, care about their needs, and support them. Fourth, the patient’s life experience is dynamic, and these oppositions can also be experienced within the individual at different times. This suggests that healthcare providers need to keep a long-term eye on patients’ lives after CIED implantation. The following subsections provide specific recommendations tailored to patient needs to improve patient quality of life.

### 4.1 Accelerate the Technological Innovation and Clinical Application of CIED

The results of this study show that while CIED helps patients fight the disease, it also brings physical discomfort to patients. Therefore, it is necessary to reduce the physical discomfort caused by CIED to patients by reducing the size of the device, reducing the number of unnecessary electric shocks, and extending the battery life of the device. The reason is, the size of the device affects the degree of foreign body intrusion that the patient feels [18], while unnecessary electric shocks can cause anxiety and pain, and even lead to patients giving up using their CIEDs [31]. Extended battery life can reduce the number of battery replacements, the risk of infection and device damage or failure, and treatment costs [31]. Regarding how to improve the patient’s acceptance of the device size, previous studies have shown that it can be achieved by optimizing the design so that the skin pressure is evenly distributed on the surface [32]. Regarding how to reduce unnecessary electric shocks, studies have shown that it can be achieved through antitachycardia pacing (ATP) [33]. ATP is an effective method for reducing electric shock and can prevent more than 50% of electric shock-induced pain [33]. In particular, intrinsic ATP (iATP) can provide adaptive ATP therapy that responds to the tachycardias of individual patients, terminating 87.8% of ventricular tachycardial episodes [34]. Regarding how to prolong battery life, studies have shown that using high-density integrated circuits and low-energy Bluetooth transmission can reduce quiescent current consumption by 9% [31]. Using chemical techniques can increase the battery capacity by 14% without a concomitant increase in battery size [31]. However, whether these achievements can play a role in the clinic and be widely applied still needs to be determined through continuous and comprehensive equipment monitoring and multi-center clinical trials. Additionally, developing industry-wide standards for predicting and reporting battery life and current drain measurements remains a formidable challenge, and meeting this challenge requires accelerated technological innovation.

### 4.2 Build a Diversified Support System

The results of this study show that patients have a strong need for external support after CIED implantation. Accordingly, it is necessary to build a support system led by medical staff, with the participation of peers, family members, and society, to help patients better adapt to life post-implantation. Both our and previous studies have shown that during office visits, medical staff needs to strengthen communication with patients to increase patient trust [11, 14]. At the same time, consider individual differences among patients, and provide patients with life guidance and information on exercise rehabilitation, driving restrictions, and equipment discontinuation to enhance the self-management capabilities of patients after CIED implantation [11, 14, 17, 35]. Improved patient outcomes have been associated with positive psychological constructs. For patients with anxiety, fear, and self-doubt, psychological intervention methods, such as cognitive behavioral therapy, quality-of-life therapy, and mindfulness therapy, can effectively promote positive emotions and improve their psychological status [36, 37, 38]. This is conducive to helping patients correctly understand themselves and face the future positively. Simultaneously, setting up support groups can strengthen communication among patients and help them obtain peer support [23]. Therefore, outside of visits, patients need to actively cooperate with their healthcare provider’s psychological interventions and participate in support groups. In addition, caregivers may lack confidence due to a lack of care experience, and medical staff also need to provide them with personalized guidance to improve the care level, thereby promoting patient recovery [39]. Family members should also avoid overprotecting patients and encourage them to actively participate in family activities and take on family responsibilities within their abilities, thereby helping them to regain their sense of value in family roles. Society needs to be more accepting of these patients. In addition, patients must be encouraged to actively participate in social activities, given re-employment opportunities, and helped return to society. Last but not least, healthcare providers can use follow-up visits to see if the patient’s support needs are being met and what needs to be improved.

### 4.3 Improve CIED Remote Monitoring

The results of this study show that the uncertainty of CVD prognosis results in a strong demand for continuous care after discharge [12]. Studies have found that CIED remote monitoring can not only help patients detect arrhythmias and equipment failures in time [40] but also quickly detect electric shock events and clinical adverse events [41]. This not only improves patient safety, reduces the number of hospitalizations [42], reduces all-cause hospitalization and mortality rates [43], but also reduces the uncertainty of CVD prognosis [40, 41, 42, 43]. Remote monitoring has been practiced and adopted to varying degrees for decades, but there is much to be improved in terms of patient access and needs. Chew* et al*. [44] (2022) refer to despite expert recommendations advocating the use of remote monitoring of cardiac implantable electronic devices, implementation in routine clinical practice remains modest due to inconsistent funding policies across health systems. In addition, Daley *et al*. [45] (2020) refer to patients who either do not have access to remote CIED monitoring data or require professional assistance in reading and interpreting the data. Fraiche *et al*. [46] (2021) refer to patients who also want the ability to integrate individual preferences into remote monitoring alarm systems so that they can choose how the alarm is handled for a better experience. Accordingly, it is necessary to increase financial investment to improve the reimbursement system, develop patient-centered remote monitoring applications, and carry out remote monitoring education and training for patients, to meet their needs for self-health control.

## 5. Limitations

Our meta-synthesis has certain limitations. Firstly, following completion of the literature review, the studies which were included were qualitative studies with limited numbers of participants, cumulatively from all eighteen studies, 301 patients with CIED were involved. Understandably, the number of patients cannot be considered representative of all patients with CIED. Secondly, this study explores the lived experience of CIED patients, but CIED patients can be divided into PM patients, ICD patients, CRT patients, *etc*. The main problems faced by different types of patients are different, and their life experiences are also different. In future studies, we hope to gain an in-depth understanding of the experiences of different types of patients from different perspectives and combine the results with those of this study to provide patients with more precise intervention strategies and guidance, thereby helping them to quickly adapt and effectively cope with life after CIED implantation.

## 6. Conclusions

In this study, we carried out a meta-synthesis of qualitative research on the life experience of patients after CIED implantation, and systematically analyzes the status quo and existing problems of patients after CIED implantation in terms of equipment symbiosis, external support, and self-coping, which to a certain extent reflects the postoperative life experience of patients. Our findings highlighted the need to accelerate the technological innovation and clinical application of CIEDs; build a diversified support system led by the medical system but with the participation of family members, peers, and society; and improve remote monitoring of the devices to help patients improve their quality of life.

## Supplementary Material

Supplementary material associated with this article can be found, in the online version, at https://doi.org/10.31083/j.rcm2410304.
